# Randomization techniques for assessing the significance of gene periodicity results

**DOI:** 10.1186/1471-2105-12-330

**Published:** 2011-08-09

**Authors:** Aleksi Kallio, Niko Vuokko, Markus Ojala, Niina Haiminen, Heikki Mannila

**Affiliations:** 1 Research Environment Services, CSC - IT Center for Science Ltd., P.O. Box 405, Espoo, 02101, Finland; 2 Department of Information and Computer Science, Aalto University, P.O. Box 15400, Espoo, 00076, Finland; 3 Department of Computer Science, University of Helsinki, P.O. Box 68, Helsinki, 00014, Finland; 4 Data Analysis Research Programme, Helsinki Institute for Information Technology HIIT, Helsinki, Finland

## Abstract

**Background:**

Modern high-throughput measurement technologies such as DNA microarrays and next generation sequencers produce extensive datasets. With large datasets the emphasis has been moving from traditional statistical tests to new data mining methods that are capable of detecting complex patterns, such as clusters, regulatory networks, or time series periodicity. Study of periodic gene expression is an interesting research question that also is a good example of challenges involved in the analysis of high-throughput data in general. Unlike for classical statistical tests, the distribution of test statistic for data mining methods cannot be derived analytically.

**Results:**

We describe the randomization based approach to significance testing, and show how it can be applied to detect periodically expressed genes. We present four randomization methods, three of which have previously been used for gene cycle data. We propose a new method for testing significance of periodicity in gene expression short time series data, such as from gene cycle and circadian clock studies. We argue that the underlying assumptions behind existing significance testing approaches are problematic and some of them unrealistic. We analyze the theoretical properties of the existing and proposed methods, showing how our method can be robustly used to detect genes with exceptionally high periodicity. We also demonstrate the large differences in the number of significant results depending on the chosen randomization methods and parameters of the testing framework.

By reanalyzing gene cycle data from various sources, we show how previous estimates on the number of gene cycle controlled genes are not supported by the data. Our randomization approach combined with widely adopted Benjamini-Hochberg multiple testing method yields better predictive power and produces more accurate null distributions than previous methods.

**Conclusions:**

Existing methods for testing significance of periodic gene expression patterns are simplistic and optimistic. Our testing framework allows strict levels of statistical significance with more realistic underlying assumptions, without losing predictive power. As DNA microarrays have now become mainstream and new high-throughput methods are rapidly being adopted, we argue that not only there will be need for data mining methods capable of coping with immense datasets, but there will also be need for solid methods for significance testing.

## Background

*Randomization methods *are techniques for significance testing that are based on generating data that shares some of the same properties with the real data, but lacks the structure of interest. For example, if we are interested in predicting a target variable on the basis of some explanatory variables, then we can randomize the target variable to remove any real connection between the explanatory and target variables. The prediction method is run on randomized data, and the accuracy of the resulting classifier is noted. This is repeated for, say, 10000 randomizations, and the accuracy of the classifier obtained on real data is compared with the results on randomized data to obtain an empirical *p*-value. See [1] for an overview on using randomization methods for significance testing.

A randomization method is based (explicitly or implicitly) on a null model, *i.e*., a description of what the data would look like in the absence of the pattern of interest. In the example above, the null model states that the data looks like the original data, except that the target variable is random (but has the same distribution of values as the original one). A well-studied example of a null model is in the context of 0-1 matrices, where one can consider the class of matrices having the same row and column sums as the original data [2-4]. In the realm of gene expression data, 0-1 matrices can be produced by discretizing data into differentially and non-differentially expressed values. Using the null model to maintain the number of 1s in the columns and rows in significance testing tells whether the data analysis result is caused just by the row and column sums, *i.e*., the count of differential expression values for genes and samples.

Permutation testing has been widely used in biological studies, as it is a natural fit with comparative clinical trials (see [5-9] for examples). Straightforward permutation methods have, however, a limited scope, but a larger variety of problems can be tackled by using computationally more advanced methods. Advanced methods, *e.g*. Markov-Chain Monte Carlo based algorithms, have had success in fields such as ecology [3,10,11]. Ecological data cannot in most situations be produced using statistically controlled procedures such as replicates and comparing experimental samples to control samples. In molecular biology similar challenges are faced especially when using high-throughput measurement instruments. As an example, null models have been used in determining the significance of co-occurrence patterns in studying potential transcription factor binding sites [12] or generic time-dependent variation of gene expression [13].

Nowadays vast amounts of data are produced by using microarrays, with the intent of detecting complex patterns such as periodic gene expression. Periodic expression is central in many fields, *e.g*., in cancer research and in circadian rhythm research. In analyzing possible periodicity of gene expression, testing the significance of the results is quite difficult: As there are thousands of genes, it is likely that at least some of them will show periodic behavior by chance. In general high-throughput exploratory methods such as DNA microarrays are very prone to misinterpretation [14].

Gene periodicity studies measure expression for a large set of genes in a series of time steps, covering typically one or more assumed gene cycles. Due to practical reasons sampling interval is often long and a control experiment repeating the same measurement on non-induced cells has not been performed. Microarray measurements are laborious and expensive, limiting the amount of samples that can be made. Emerging RNA-seq methods are poised to replace expression microarrays, but for the foreseeable future the amount of samples is equally limited with them.

There has been discussion on the true interpretation of gene periodicity results since the first two experimental yeast studies [15,16]. The first studies suggested that 400 to 1000 genes are periodically expressed. However most notably Cooper *et al*. have suggested that data produced in the yeast studies does not warrant such strong conclusions about gene periodicity [17]. They stress the importance of careful data analysis and interpretation, calling for new methods of significance testing.

Methodological research in periodicity studies has concentrated on producing alternative approaches for identifying periodicity in gene expression time series. Proposed methods vary from simple Fourier analysis to more elaborate mathematical and statistical analyzes [18]. As it is often in rapidly developing fields, computational methods for verification of results are developed more slowly. There has been no critical studies on methods of periodicity significance testing, until the interesting study by Futschik and Herzel [19]. They proposed a *background model *of gene expression based on autocorrelative random processes. Using the background model they were able to improve the quality of significance detection, compared to random permutations or Gaussian models. An important aspect of the Futschik and Herzel study was that they discussed the assumptions and justifications of their background model, which had not been done in previous work. Obviously a single study can cover the area only partially and the important significance testing question should be studied further. In biomedical sciences, periodicity studies have continued since, with the focus moving from gene cycle to circadian cycle (*e.g*., [20-22]). However on the method development arena new developments have been scarce.

In this study we present null models for significance testing. The null models are formulated as randomization methods, *i.e*., algorithms for generating datasets. Generated randomized datasets share certain characteristics with the original dataset, so that they are realistic samples for calculating how extreme values of periodicity we should observe by chance. First we define the randomization methods and describe how they can be applied to periodicity analysis. Next we review the differences in the results produced by different methods, and last we discuss the implications of our results for microarray studies and other fields of high-throughput biosciences.

## Methods

We present the significance testing approach and the randomization algorithms to be used within the framework. First we describe how periodicity can be measured from gene expression and how measured periodicity is interpreted statistically.

### Measuring periodicity

As a measure of gene periodicity we use a simplified version of the *Fourier score *approach used by [15], based on a Fourier analysis of the gene expression time series. While several different approaches to periodicity detection have been discussed in the literature, the original Fourier score approach is the most commonly used and also was found to perform better than more intricate methods [18].

We consider time series matrices produced by measurements of gene expression at consecutive time points. Let the gene expression matrix *E *have *n *rows and *m *columns, corresponding to *n *genes and *m *sequential time points. The matrix has been produced by measuring expression of each gene at uniform intervals during *c *gene cycles.

Periodicity score *F *for gene *g *at frequency *w *is defined in Equation (1). Here *E*(*g*, *t*) is the normalized expression value of gene *g *∈ {1, ..., *n*}, at time *t *∈ {1, ..., *m*}.(1)

For normalization we use the same procedure as Futschik and Herzel [19]. First missing values are imputed with KNN-imputation. Then each row is shifted to have mean 0 and scaled to have standard deviation 1. These normalization steps are done to original and randomized data before calculating periodicity scores [19].

### Detecting significant periodicity: empirical *p*-values

Periodicity scores allow us to compare periodicity levels between genes. To identify genes that are to be considered periodic, we need a way to decide which periodicity scores are significantly large. In the original studies periodicity was decided by using a threshold for the score [15,16]. Threshold is selected in an *ad hoc *manner by looking at some characteristics of the dataset, and selecting a value that gives results that are considered to be reasonable.

Using a fixed periodicity score threshold does not give us any approximation on how likely it is to detect the given level of periodicity by pure chance. For estimating how unlikely a periodicity score is, we compare it against the distribution of scores that we would probably see when only non-induced genes are measured, *i.e*., against the null distribution. *p*-value is derived by comparing score in the original dataset to the null distribution. To define and estimate the null distribution, we use randomization based null models that are described later.

Let *F*(*x*) be the periodicity score of row *x*, let *g *be the original row and let  be the *R *randomized rows. Empirical one-tailed *p*-value for the periodicity of row *g *is defined by the Equation (2).(2)

If the empirical *p*-value is small, then we can conclude that under the null model it is unlikely that the observed periodicity of *g *is a product of luck. However when multiple observations are made, the probability of detecting periodicity by chance increases. Therefore we need to employ multiple testing correction. The traditional way to handle multiple testing correction is to control *family-wise error rate *(FWER), using for example Holm-Bonferroni method [23]. FWER is typically considered too strict for DNA microarray studies [24,25]. It controls the probability of making even a single error, resulting in very narrow rejection regions. Microarray studies increasingly use less strict approach of controlling *false discovery rate *(FDR), *i.e*., the proportion of mistakes, with the Benjamini-Hochberg method [26]. It will also be used here as one of the two multiple testing methods.

An important caveat of the Benjamini-Hochberg method is that it controls the *expected rate *of false discoveries and can have suboptimal performance in permutation testing [27]. As a randomization based null model can be readily used to generate either cyclicity scores from the null distribution or empirical *p*-values, majority of available multiple testing approaches can be used. Unfortunately the more advanced solutions, such as those from [28] or [29], are more complicated. As multiple testing control methods are not the focus of this study, we use the more simplistic Benjamini-Hochberg method.

### Detecting significant periodicity: Futschik and Herzel threshold

Futschik and Herzel use an alternative approach to significance testing [19]. They do not calculate empirical *p*-values, but instead use the raw periodicity scores produced by the randomization methods. Equation (3) below defines the Futschik-Herzel empirical false discovery rate *FH*(*f*) for threshold *f*. The equation is based on periodicity scores in randomized data *F*(*g*, *r*) and on periodicity scores in the original data *F*(*g*). Count of scores greater than or equal to *f *are calculated over all genes *g *∈ {1, ..., *n*} and randomized matrices *r *∈ {1, ..., *R*}, where *R *is the number of randomized matrices produced.(3)

Here  is one when the condition *P *is true, and zero otherwise. By using Equation (3) it is trivial to select *f *so that *FH*(*f*) = *q*, where *q *is the desired false discovery rate. This approach is similar to threshold selection in [15,16], but it is important to note that Futschik and Herzel do not manually tune the score threshold *f*, but instead select *q *and derive the value of *f *by using the equation. They do not discuss the statistical justifications of the method, or specify the underlying assumptions or the exact nature of the provided control for "empirical false discovery rate".

### Randomization methods

**Figure 1:**
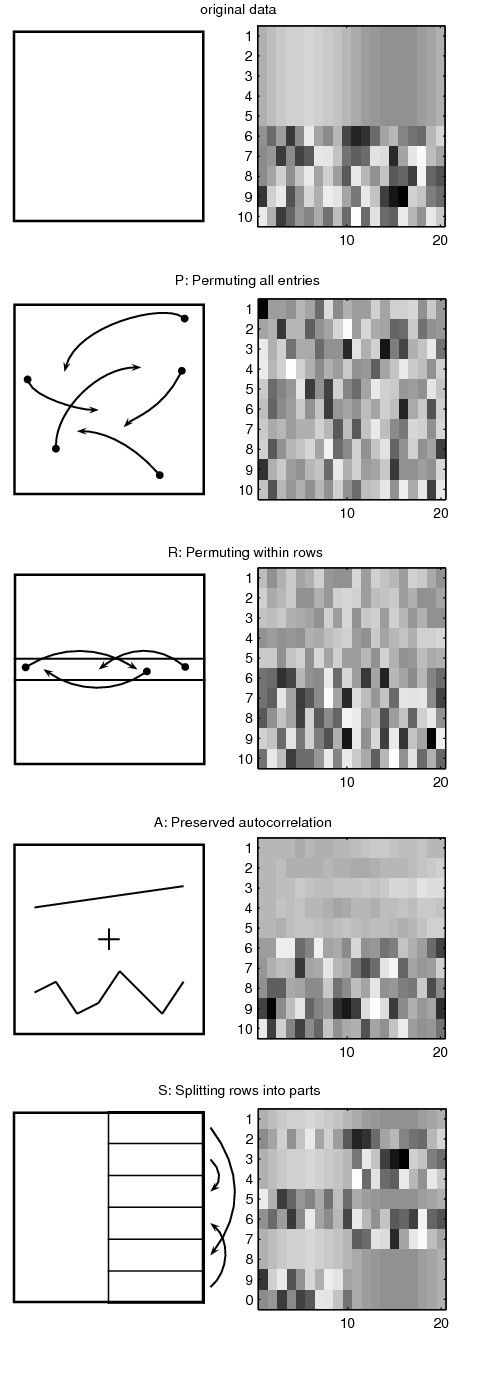
**Example dataset and results after applying randomization methods of this study**. The original dataset is a simulated expression matrix of 10 genes and 20 time points. The first 5 genes are cyclic and the values for the last 5 genes are randomly drawn from a normal distribution, as can be seen from the heatmap of the original dataset. Randomized datasets are illustrated with a simplified chart showing the basic operating principle and a result dataset shown as a heatmap.

Empirical *p*-values provide a way of using a null distribution of gene expression matrices to assess the significance of periodic signals in the original measured data. To produce null distributions of expression matrices, we describe four techniques for generating randomized *n *× *m *matrices *E*'; that share certain characteristics with the gene expression matrix *E*. The methods either use the original dataset as the data to be randomized or calculate parameters of a data generating model from the original dataset [30]. The methods are illustrated in Figure 1.

#### P: Permuting all entries

Randomized matrix  is generated by permuting the values of all entries in matrix *E*. The result  maintains the original value distribution of *E*, but does not maintain, *e.g*., the row and column distributions.

It is obvious that permutations are a drastic randomization method and do not preserve much of the structure in the dataset. They are used in periodicity studies, as in the seminal paper by Spellman *et al*., where they argue that an upper bound for false positive count can be achieved with permutation randomization [15].

#### R: Permuting within rows

Randomized matrix  is generated by permuting the values within each row of *E*. The result  maintains the original value distribution and the row value distributions. A row in  is dependent only on the corresponding row in *E*.

Spellman *et al*. argue that an upper bound for false positive count can be achieved with randomization by permuting within rows [15], so the actual false positive count would be between the amount of false positives produced by methods *P *and *R*. Our results indicate that both of the methods should be considered optimistic: They produce only loose lower bounds for the number of false positives.

#### A: Autocorrelation

Cell cycle time series data often exhibits autocorrelation due to the procedure employed to bring cells to a synchronized state in the beginning of the time series. In a method proposed by Futschik and Herzel gene expression is modeled as an *autoregressive AR*(1) *process *for which the value of expression at time *t *depends linearly on expression at time *t - *1 up to a normally distributed random variable [19]. Specifically, *X_t _*= *a*_1 · _*X_t_*_-1 _+ *Z_t_*, where *X_t _*is the time dependent random variable, *i.e*., gene expression, while *a*_1 _is the first order autocorrelation of *X_t _*and *Z_t _*is the independent normally distributed random variable.

As *Z_t _*is a random variable, we can easily calculate *a*_1 _and the variance of *Z_t _*from the original data matrix *E *and use them to generate a random matrix  with the same autocorrelation and variance of *Z_t_*. Yang and Su have suggested using multiple methods simultaneously for estimating autocorrelation coefficients of circadian gene expression data [31]. In our experiments we did not see non-marginal differences when using other coefficient estimators and for that reason used the Yule-Walker method that was also used by Futschik and Herzel [19].

One aspect of the Futschik and Herzel approach is that variance of the whole row is normalized, but the data generating model preserves only variance of the normally distributed random variable *Z_t_*. With datasets of this study, we typically see variances less than the original in randomized data. When variance is normalized after randomization, the data is effectively scaled up on average. As Fourier score is used for periodicity detection, also the periodicity scores are scaled up. Unlike the other methods,  does not maintain the original value distribution of the matrix elements. For a detailed description of the method we refer to supplementary materials of the original article and especially to the related R-package [19,32].

#### S: Splitting rows into parts

We propose a new randomization method that takes a gene cycle and splits it into two equal sized parts referred to as the prefix and the suffix. Then we simply randomize the connection between prefixes and suffixes, *i.e*., each suffix is randomly assigned to follow a prefix.

More formally, assuming there is only one gene cycle, a row *g *in the randomized matrix  with *m *time points is generated as

Here index *h *is chosen at random without replacement from {1, ..., *m*}. In the formula, we combine rows *g *and *h *in the original data to produce a randomized row. When there are multiple gene cycles, we split each cycle into prefixes and suffixes. Then again, we combine the prefixes of row *g *with suffixes of row *h *to create a new row. Each row in the randomized data contains values from two rows in the original data, except for the rare case that *g *= *h*, when the randomized row is actually equal to a one row in the original data.

#### Interpreting the null models

We motivate our randomization method by interpreting the methods as null models that produce random samples from a null distribution. What we would like to get are samples from a situation where the same genes are measured under the same circumstances, but without a treatment that is assumed to induce cyclic expression.

The randomization method *P *can be interpreted as sampling each expression value from a global null distribution. The strength of the method is that there are a large amount of individual values in a microarray experiment and thus we have a robust sampling distribution. However by sampling each value independently we completely eliminate dependencies between values of a single gene. In time series data consecutive values are naturally strongly dependent and a realistic null model cannot be produced without taking these dependencies into account.

In expression data there are differences between the average level of expression of different genes, but complete permutation makes data uniform. The method *R *improves on *P *in this aspect: now we sample each gene from its own distribution, preserving differences between average levels of expression of the genes. Unfortunately, there are very few values per gene and building a sampling distribution from, say, 20 values is not robust. Besides that, the distribution of values for induced and non-induced genes can be very different, and permuting expression values of an induced gene does not necessarily produce an expression profile that a typical non-induced gene might have. Dependencies between values of consecutive time points for a single gene are not taken into consideration with *R*, either.

It is possible to sample genes by taking their average level of expression and adding normally distributed noise, with the same variance than in original data. This approach assumes that non-induced genes produce constant level of expression, excluding the noise. However processes not related to cyclicity can produce varying expression patterns that are not captured in this model. The randomization method *A *takes into account some of the patterns by adding *AR*(1) autocorrelation to the model. *AR*(1) autocorrelation can capture certain stress response patterns that the treatment induces and are argued to be a major feature of gene cycle data [19]. Formulation of *A *does not, however, preserve the average level of expression for each gene, but the level is mostly decided by the rather arbitrary first measurement value. Also it is not known how well stress response is modelled by *AR*(1) autocorrelation: The model can only capture a linear pattern that covers the whole timeseries, but the actual stress response pattern might be non-linear and only cover the first timepoints of the dataset. An open question is also what other patterns of non-induced expression should be taken into account.

As can be seen, producing a general null model of gene expression is very difficult. And what is more important, development of null models has been progressing from simple to more advanced. Each new feature that is taken into account can make the model more realistic, but we do not know when the model is realistic enough. The idea of significance testing with *p*-values is to assume the absence of the effect or pattern of interest (null hypothesis), to gather evidence to invalidate null hypothesis in favor of an alternative hypothesis and to assess the probability that the evidence was a product of luck. As null hypothesis is the starting assumption and we are producing evidence against it, data should be analyzed in a conservative way. If there is a bias in our methods, it should be towards conservative interpretation. A null model that exaggerates the randomness of the null distribution clearly contradicts with the statistical framework.

Method *S *maintains the original value distribution, value distributions of each column, and also the correlations between columns inside prefixes and inside suffixes. The method is based on the assumption that many of the genes are non-induced and hence the distribution of each column is close to the distribution of a non-treated sample. We take into consideration that data can contain global patterns such as stress response. Values are not mixed across timepoints and hence global stress response patterns are not removed, but experiment wide cyclic structures are. The method can produce a small amount of cyclic genes to the randomized data by combining two cyclic genes, with matching phases, in the original data (or a cyclic gene with itself), which produces a slight conservative bias.

We point out that method *S *does not take into account the known fact that there are correlations between genes. However our model is a conservative null model for gene periodicity detection: Correlations do not play a role when the periodicity of each gene is measured independently. If we would be assessing the significance of, say, clustering, then correlations should be taken into account.

### Analysis of periodicity score distributions

This section analyzes the theoretical properties of the randomization methods. Reading this section is not necessary for understanding the rest of the article.

To better understand results produced by different randomization methods, we investigate the theoretical properties of the periodicity scores produced by them. This analysis shows clearly how the commonly used randomization methods cannot take the time component or the sampling frequency of periodicity data into account even though the methods are used for analyzing exactly the time-based structure of the data.

A perfect cyclical gene has values  for *t *= 0,1,2, ..., *m *- 1; let *c_k _*= (*c_k_*_0_, *c_k_*_1_, ..., *c_k_*_(*m*-1)_). Denoting by *E *= (*e_gt_*)*_n × m _*a dataset, and by *E_g _*the *g*th row of *E*, let *E_g _· c_k _*be the dot product of the vectors *E_g _*and *c_k_*. Consider the slightly modified *k*th periodicity score for the *g*th row *E_g _*of *E *defined by

We use this modified version of the periodicity score to make the results more easily understandable while still retaining all the important characteristics of the metric. The periodicity score of *c_k _*is . Therefore periodic genes receive the same score regardless of the data size and sampling frequency.

By straightforward calculations based on these definitions it is possible to analyze the periodicity scores given by each of the randomization methods. In the following we express the expectations and variances of periodicity score distributions. Details for the results can be found in the Additional file 1.

#### Periodicity scores when randomizing by elements

For the *P *method that permutes all the data elements at random the expected value and variance of the periodicity score for any gene in the randomized data are

In the equations  and  denote the average values of matrix *E*'s elements raised to power 2 and 4 respectively. The statistics of the values generated by the *P *method do not have any connection to the time component of the data, but only to the value distribution of data. This can be restated so that the method simply calculates the whole signal energy (*i.e*., sum of squares of elements), with noise included, contained in the data set. However only the fluctuation corresponding to some cyclical structure should be accounted.

**Figure 2:**
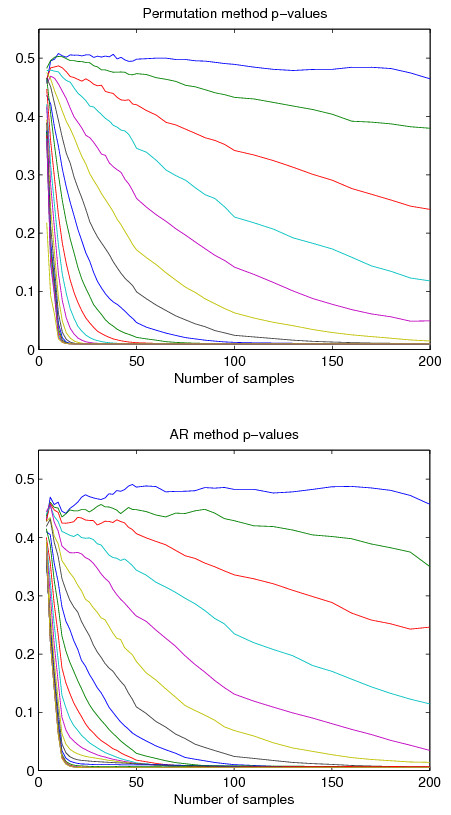
**Examples of how the number of samples affects the *p*-values reported by the permutation methods *P *and *R *and by the autocorrelation method *A***. In both figures, each separate curve corresponds to a standard sine wave at interval [0, 4*π*], with noise added so that the sine signal accounts for 5%, 10%, ..., 100% of the total signal energy. The slight bumps in the curves for method *A *correspond to the cosine in the denominator of Equation (4).

It can also be seen that as the number of samples in data *m *grows, both the statistics become smaller and smaller. This is true, however, only if the data contains no cyclical components. In crude terms, any actual cyclical structure in data supports the periodicity score and prevents it from sinking significantly lower than some given level as *m *grows. Therefore, as seen in Figure 2, the *p*-values for data with cyclical structure move consistently toward zero as *m *grows.

For the *R *method that randomizes each row separately we reach similar results. The statistics of the periodicity scores of the randomized data are

(These values are across all the genes, for gene-wise values simply fix the value of *g*.) Here again, the statistics do not depend on anything except the value distribution of the data without any regard to the order or placement of the elements. These results on the theoretical properties of the *P *and *R *methods make them questionable if any consideration should be given to the actual dataset or the purpose of the experiments.

#### Periodicity scores produced by A: Autocorrelation

In the randomization method of [19] the values of a data row (*X_t_*)_1 × *m *_are generated as values of a martingale: *X*_0 _= *Z*_0_, *X*_1 _= *αX*_0 _+ *Z*_1_, *X*_2 _= *αX*_1 _+ *Z*_2_, ..., *X_m_*_-1 _= *αX*_*m*-2 + *Zm*-1_. We require that |*α*| < 1 and the *Z_t _*are i.i.d. samples from some distribution  with . Let us present the data signal as its Fourier decomposition, *i.e*., as a sum of different cyclical sine signals . We may then calculate the autocorrelation of the signal to equal

Here the values *α_k _*give the amount of cyclicity the signal has for each different frequency. The expectation and variance for periodicity scores of data samples generated with this autocorrelation method of [19] can be shown to be(4)

In the equations the notation  means that the error term has absolute value at most some constant times *f*(*m*).

The periodicity scores of the generated samples depend on  since the more the data fluctuates the higher is the variance  chosen in the method and the more probable it is for any generated sample to receive high periodicity score. Furthermore the time component and the periodicity of the original gene data has influence on the samples' periodicity through the autocorrelation measure *α*. To further simplify the equations one can note that for a strongly periodic gene its *α *is practically equal to for  some single *k*.

The statistics depend on *m *the same way as for methods *P *and *R*, decreasing as the number of samples grows, because for higher sampling frequencies the data fluctuates rather slowly (with *α *≈ 1) and the high frequency noise is more easily distinguished. This also exemplifies how *A *measures the significance of gene cyclicity against a background of pure noise. Figure 2 illustrates the behavior of the *p*-values with increasing sample counts.

#### Periodicity scores produced by S: Splitting rows into parts

We now make an assumption that cyclical signals are cyclical in all parts of the signal, without turning into pure noise at any point. If we denote by *α_gk _*the *k*th Fourier coefficient of the *g*th gene, *i.e*., the *g*th gene has Fourier decomposition , then we can express the periodicity score statistics as

These values indicate that the cyclicity of the genes on each of the frequencies gets randomized separately, since the statistics depend only on the current value of *k*. On the contrary to the case of methods *P*, *R *and *A*, the statistics do not diminish with longer sample sizes or higher sampling frequencies. This happens, because the *S *method focuses on detecting genes that are exceptionally cyclical in the context of all the genes in the given dataset, not against an absolute background of pure noise as the other methods do.

#### Summary of analysis

We illustrated how *S *is the only method using the background distribution of the analyzed genes instead of a pure noise background. We also showed how the behavior of *P*, *R *and *A *changes with an increase in the sample count. Methods *P*, *R *and *A *suffer from high noise levels in data whereas *S *dissects the data and considers each frequency separately, which is also how the actual test score is calculated. Finally the *P *and *R *methods cannot even be seen as actual periodicity tests since they ignore any periodicity in the data and redistribute only the signal energy of the data, no matter whether it arises from periodicity, other meaningful patterns or from random noise.

## Results

In the previous section we analyzed the four different randomization methods discussed in this manuscript. The analysis showed that the method *S *has sound theoretical foundation for assessing the significance of periodicity. In this section we will back analytical results with experiments. We will also compare two different multiple hypothesis testing approaches and discuss their differences.

All methods were implemented with Matlab and experiments were run with a standard workstation PC. Matlab code is available from a companion web site at http://www.cs.helsinki.fi/u/akallio/periodicity/.

### Datasets

Results in rest of this section are based on gene expression data from well-known yeast gene cycle studies [15,16]. The goal of those studies was to identify the subset of genes that are cyclically expressed. For comparison we include a different type of dataset where majority of the genes show strong cyclic pattern, from a malaria protozoan study [33]. A summary of the datasets and their characteristics is presented in Table 1.

**Table 1:** Characteristics of the datasets, including reported and computationally detected numbers of cycles

Dataset	Citation	Reported gene cycles	Detected cycles	Genes	Timepoints
ALPHA1C	[15]	1	0.81	6178	9
ALPHA2C	[15]	2	1.98	6178	18
CDC15	[15]	3	2.19	6178	24
CDC28	[16]	2	1.88	6178	17
ELU	[15]	1	0.96	6178	14
MALARIA	[33]	1	1.06	4221	46

The dataset ALPHA2C has been produced by bringing the whole yeast cell culture to a hypothetically synchronized state using *alpha-factor *arrest. The dataset ALPHA1C is a version of ALPHA2C with only the first cycle included (restricted to first 9 time points), mimicking a very low quality dataset. Datasets CDC15 and CDC28 are based on temperature arrest of *cdc15 *and *cdc28 *temperature sensitive mutants. Contrary to the other yeast datasets, the dataset ELU is based on elutriation where instead of external treatment a subset of hypothetically synchronized cells was collected from the population. The dataset MALARIA is based on a synchronized *in vitro *culture.

We could use the hypothetical numbers of gene cycles reported for each of the datasets, but the accuracy of periodicity calculation can be improved by detecting the number of cycles from the dataset [15]. For that we find *c_adj_*, where 0 <*c_adj _*≤ 2*c *and *c *is the hypothetical number of cycles. We iterate with 0.01 increments and choose *c_adj _*so that it maximizes the sum of periodicity scores for all genes. Chosen values of *c_adj _*are reported in column Detected cycles of Table 1.

### Comparison of randomization methods

As our initial test of the methods, we studied the behavior of the periodicity score on randomized data. We computed the scores for each row in each dataset, and selected for each dataset three thresholds *δ_s _*so that a fraction of *s *= 0.05, *s *= 0.10, or *s *= 0.20 of the genes have scores higher than *δ_s_*.

For each dataset and each method we then generated 100 randomized datasets, and computed the fraction of randomized data rows that have periodicity scores higher than the threshold. If there is no periodic structure in the data, the distribution of the periodicity scores in the randomized data should be about the same as in the original data, and hence the fraction of randomized rows with scores above *δ_s _*should be about *s*. On the other hand, if the data has strong periodic structure, the distribution of the scores of the randomized rows should be clearly different from the distribution of the scores in the real data, and the fraction of genes with scores above *δ_s _*should be less than *s*. The ratio of randomized genes labeled as periodic is reported in Table 2 for each combination of dataset and randomization method.

**Table 2:** Proportion of genes labeled periodic in the randomized data

Dataset	*P*	*R*	*A*	*S*
	*s *= 0.05			

ALPHA1C	0.005	0.004	0.015	0.050
ALPHA2C	0.003	0.002	0.009	0.015
CDC15	0.001	0.001	0.005	0.013
CDC28	0.001	0.001	0.010	0.018
ELU	0.001	0.001	0.018	0.050
MALARIA	0.000	0.000	0.002	0.050

	*s *= 0.10			

ALPHA1C	0.015	0.013	0.036	0.100
ALPHA2C	0.014	0.013	0.031	0.048
CDC15	0.007	0.007	0.024	0.047
CDC28	0.009	0.008	0.037	0.055
ELU	0.003	0.002	0.035	0.100
MALARIA	0.000	0.000	0.004	0.100

	*s *= 0.20			

ALPHA1C	0.048	0.044	0.086	0.200
ALPHA2C	0.059	0.056	0.089	0.125
CDC15	0.047	0.045	0.083	0.133
CDC28	0.047	0.044	0.118	0.147
ELU	0.011	0.009	0.074	0.200
MALARIA	0.000	0.000	0.010	0.200

Periodicity is determined by thresholds *δ_s _*determined so that a fraction of *s *of the genes in the original dataset have scores above *δ_s_*.

Table 2 shows that methods *P*, *R*, and *A *consider that the fraction of genes in the randomized data with high periodicity scores is much lower than in the real data, for all datasets. That is, these methods would indicate that all datasets have significant periodic structure.

On the other hand, results for method *S *are different for different datasets: for datasets ALPHA1C ELU, and MALARIA the fraction of genes with high scores is the same in the randomized data and in the real data, while for ALPHA2C, CDC15, and CDC28 the randomized datasets have fewer genes with high scores. Thus method *S *indicates that only in the datasets ALPHA2C, CDC15, and CDC28 there is clear evidence of periodicity. These assessments produced by method *S *are consistent with the previous results. ELU was found to be low quality already by the original authors [15]. ALPHA1C was artificially constructed to be an example of a dataset with too few timepoints for reliable periodicity detection. The dataset MALARIA can be seen to be a special case, and it is discussed next.

### Limitations for generating non-induced expression

The MALARIA dataset is an example of an atypical microarray dataset and it is not a good fit for randomization methods presented in this study. This is because we are measuring here the count of genes that have significantly higher periodicity scores than given by the null model. MALARIA dataset contains mostly periodic genes, so it does not contain much information about gene expression of other types than of the synchronized periodic pattern. Simple permutations *P *and *R *do not preserve much of the structure of the original dataset and compare strongly periodic original dataset to noise mostly, resulting in no periodic genes to be detected in the randomized data. Method *A *is based on the assumption of Gaussian expression with *AR*(1) autocorrelation, and does not preserve much of the structure in the original data either. As was previously shown in the analysis, method *S *is different in this regard. It uses the dataset to build a model of all expression patterns without other major assumptions about the data, and as the dataset is very biased, so is the model. *S *reports that there is no periodicity in the data that cannot be explained by chance alone.

The advantage of microarray experiments is that if we are studying the behavior of maybe some dozens of genes, the dataset will contain hundredfold of non-induced genes to compare to [34]. As was shown in our analysis, the method *S *can be used to detect genes that are exceptionally periodic. However, the assumption about large numbers of non-induced genes is not true for MALARIA. For that reason we do not consider any of the results for MALARIA realistic, but hold the view that the recommended solution would have been to conduct more measurements to get data on typical expression patterns in the case of no treatment. If such data is not available, then additional assumptions have to be done to roughly approximate the null distribution of the periodicity score. Assumptions behind the method *A *are not realistic in this case. Level of autocorrelation and variance of Gaussian distribution are calculated from data, which now contains mostly induced genes. Therefore calculated parameters are not necessarily realistic for non-induced genes.

### The number of periodic genes

To estimate the number of periodic genes in the datasets, we use the above randomization methods to produce randomized samples and apply both the Benjamini-Hochberg procedure on empirical *p*-values and the method of Futschik and Herzel on periodicity scores. For validation we use a benchmark gene list from [18] that presents three benchmark sets of genes for the yeast periodicity studies, corresponding to datasets from Spellman *et al*. and Cho *et al*.. The first benchmark set comprises of 113 genes that were confirmed to be periodic in small scale laboratory experiments. The other two sets are based on less decisive methods, so we restrict our comparison to the first set only.

For comparing the methods we use the positive predictive value (PPV), which was also used in [19]. For a given result list the value is defined as(5)

Here *TP *is the count of true positives, *i.e*., the number of genes that the method considers periodic and that are found from the small scale experiment benchmark set. *FP *is the count of false positives, *i.e*., the number genes that the method reports as periodic, but which do not occur in the benchmark set. It is important to bear in mind that the benchmark set does not contain all periodic genes, so the absolute value of *PPV *has little meaning, but the relative difference between values indicates a difference between the quality of the two candidate gene lists. Also for this reason only one variable should be changed at a time when doing the comparison. The *PPV *and total count of positives is reported in Table 3. As *PPV *is not robust when the number of positives is small, and undefined when there are no positives, we omit the value when there are less than 10 positives.

**Table 3:** Positive predictive value (PPV) and in parentheses the count of all positives (true and false) for the given datasets and randomization methods

Dataset	*P*		*R*		*A*		*S*	
FDR 0.01, Futschik and Herzel								

ALPHA1C		(0)		(0)		(0)		(0)
ALPHA2C	0.315	(111)	0.315	(127)		(0)		(0)
CDC15	0.199	(251)	0.197	(259)	0.400	(15)		(0)
CDC28	0.193	(145)	0.168	(202)		(1)		(0)
ELU	0.184	(103)	0.183	(104)		(0)		(0)

FDR 0.01, Benjamini-Hochberg								

ALPHA1C		(0)		(0)		(0)		(0)
ALPHA2C	0.315	(111)	0.315	(127)		(0)		(0)
CDC15	0.199	(251)	0.197	(259)	0.429	(21)		(0)
CDC28	0.193	(145)	0.168	(202)		(0)		(0)
ELU	0.184	(103)	0.181	(105)		(0)		(0)

FDR 0.05, Futschik and Herzel								

ALPHA1C	0.080	(75)	0.087	(115)		(0)		(0)
ALPHA2C	0.176	(296)	0.166	(314)	0.364	(77)	0.600	(10)
CDC15	0.140	(450)	0.136	(464)	0.215	(191)	0.429	(21)
CDC28	0.108	(435)	0.102	(500)	0.276	(58)		(2)
ELU	0.055	(1155)	0.050	(1327)		(1)		(0)

FDR 0.05, Benjamini-Hochberg								

ALPHA1C	0.077	(78)	0.087	(115)		(0)		(0)
ALPHA2C	0.176	(296)	0.166	(314)	0.372	(78)	0.667	(15)
CDC15	0.140	(450)	0.136	(463)	0.218	(193)	0.435	(23)
CDC28	0.108	(435)	0.102	(500)	0.276	(58)		(2)
ELU	0.055	(1155)	0.050	(1327)		(1)		(0)

FDR 0.10, Futschik and Herzel								

ALPHA1C	0.077	(338)	0.067	(421)		(0)		(0)
ALPHA2C	0.125	(447)	0.120	(476)	0.251	(167)	0.434	(53)
CDC15	0.107	(653)	0.105	(668)	0.188	(271)	0.292	(96)
CDC28	0.084	(651)	0.085	(685)	0.205	(132)		(2)
ELU	0.040	(2058)	0.039	(2207)		(2)		(0)

FDR 0.10, Benjamini-Hochberg								

ALPHA1C	0.077	(339)	0.067	(432)		(0)		(0)
ALPHA2C	0.125	(447)	0.120	(476)	0.253	(170)	0.434	(53)
CDC15	0.107	(653)	0.105	(668)	0.188	(271)	0.280	(107)
CDC28	0.084	(651)	0.085	(685)	0.205	(132)		(2)
ELU	0.040	(2058)	0.039	(2207)		(2)		(0)

Values are calculated using FDR levels 0.01, 0.05 and 0.10 with different control procedures.

Table 3 reports *PPV *values followed by the corresponding number of significant periodic genes in parentheses. We first compare our results to earlier results by Spellman *et al*. and Futschik and Herzel. Spellman *et al*. reported 400 to 1000 periodic genes, based on randomization methods *P *and *R*. Our results for the corresponding datasets are in the range of 300 to 450 with commonly used Benjamini-Hochberg FDR 0.05, *i.e*., much lower. As discussed earlier, Spellman *et al*. used a manually tuned threshold for periodicity scores. Their threshold does not account for the number of genes that are tested. Our framework accounts for multiple testing and produces smaller numbers of positives. If multiple hypothesis correction is omitted, our analysis pipeline with methods *P *and *R *produces approximately 1000 significant results, matching the results by Spellman *et al*..

When comparing results with those of Futschik and Herzel, we need to remember that there are differences in wavelengths that are used for Fourier calculations. We detected the number of gene cycles (or in other words wavelength) from the data, whereas Futschik and Herzel used the wavelength from the original study by Spellman *et al*.. For the Cdc28 dataset they are the same and hence our results are also practically identical to the Futschik and Herzel study. The small differences are due to different KNN-imputation implementations and slight difference in wavelenght that is not visible due to rounding. For Cdc15 the detected wavelength differs and that yields differences especially for the stricter thresholds; At FDR 0.10 they are already quite close (132 here versus 126 in Futschik and Herzel study).

When looking at FDR control parameters, the typical FDR threshold 0.05 produces the best *PPV *values, while the more relaxed FDR 0.10 is already too forgiving, at least when compared to the validation data. The FDR threshold 0.01 is very strict for this kind of study and that can be seen from the results also. There are no differences between the FDR control methods: The Futschik and Herzel method produces practically identical results to the Benjamini-Hochberg method.

The poor quality of the artificially constructed bad dataset ALPHA1C is identified by both *A *and *S *methods, but not by the simpler permutation methods. That same deficiency is even more exaggerated for the ELU dataset that was found bad in the previous section and also by Spellman *et al*. [15]: The simple methods *P *and *R *report over hundred significant genes already at FDR 0.01 and scale up to thousands at higher FDR levels. Judging by this, these two methods cannot be recommended even for simple sanity checking of the data.

For the good quality datasets ALPHA2C and CDC15, both *A *and *S *give more conservative results that have better *PPV *values. The method *S *is systematically more conservative than *A*, but also gives a better *PPV *value in every case.

**Figure 3:**
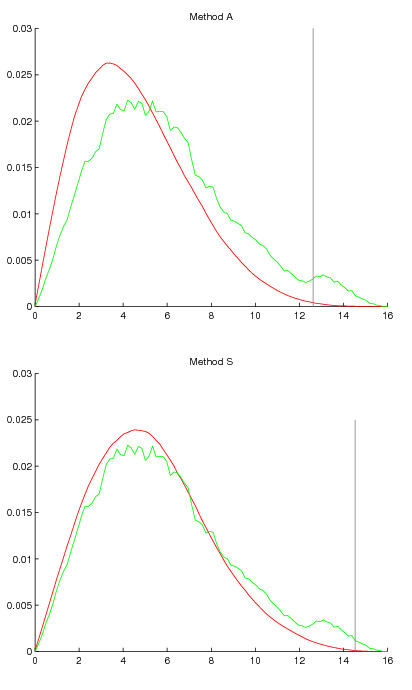
**Null distribution of periodicity scores produced by methods *A *and *S *in dataset Cdc15 plotted together with the distribution of values in the data**. Green density function shows the distribution in data and red density function shows the null distribution produced by the randomization method. The gray vertical line shows threshold of significance as decided by the Benjamini-Hochberg method at FDR 0.05.

We can examine these differences closer by looking at null distribution of periodicity scores produced by the two methods, when compared to original distribution from data. Figure 3 shows randomization generated null distribution together with distribution from data in the case of CDC15. The vertical line shows threshold of significance as decided by the Benjamini-Hochberg method at FDR 0.05. The method *S *produces a null distribution that very closely follows the distribution in original data, except naturally for the bump at the high end, *i.e*., where alternative distribution of periodic genes mixes with the non-periodic ones. Method *A *produces a null distribution that is biased towards the low periodicity scores, as the peak of the distribution is too far in the low end. In the score range 6 to 10 the null distribution drops faster than the original. This probably cannot be explained by the periodic distribution mixing with the non-periodic distribution, because it seems highly unlikely that non-trivial portions of the periodic distribution extends to that range. As the null distribution is systematically off the mark for the low and mid ranges, it does not seem plausible that *A *could be used to reliably separate the periodic and non-periodic distributions in the high range. A threshold decision based on the method *S *is the one that is supported by Figure 3.

**Figure 4:**
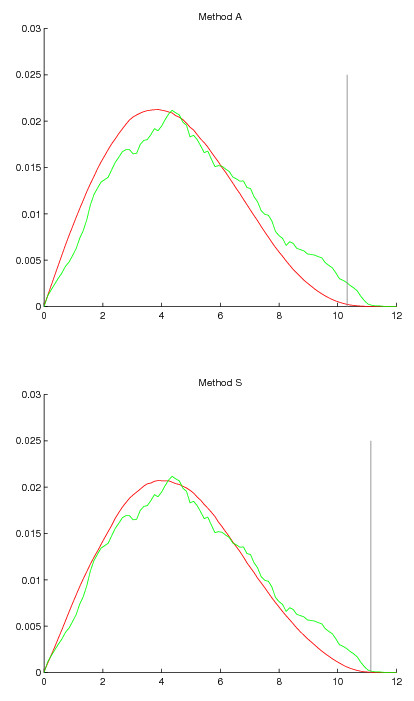
**Null distribution of periodicity scores produced by methods *A *and *S *in dataset Cdc28 plotted together with the distribution of values in the data**. Green density function shows the distribution in data and red density function shows the null distribution produced by the randomization method. The gray vertical line shows threshold of significance as decided by the Benjamini-Hochberg method at FDR 0.05.

The methods *A *and *S *disagree on the CDC28 dataset. Method *S *asserts only two genes to be significant even on the high FDR levels, while the method *A *gives more optimistic estimates. The case is analogous to dataset ALPHA2C, where both give zero significants at FDR 0.01. The only difference is that CDC28 is given more conservative assessment, to the point that *S *does not accept more than two positives at FDR 0.10. However at FDR 0.20 also method *S *would have already given 90 positives and we could more easily observe similar trend than in ALPHA2C. To examine this further, we again plot the distributions produced by the two methods in Figure 4. When compared to the dataset CDC15, the bump of periodic genes is not pronounced at all and it seems that the area where significant scores are located is populated mostly by genes from the non-periodic distribution. In this dataset method *S *still produces a distribution that more closely follows the original one, though the difference to method *A *is not as large. The peak of the distribution is somewhat too far in the low end for *A*, and perhaps slightly for *S *also. When looking at the null distribution produced by *S*, it is easy to understand why only two genes were declared significant. The two distributions of induced and non-induced genes are so mixed that reliably separating them with a threshold does not seem possible. Method *A *paints a more optimistic picture and admittedly it is not far off the mark in the distribution graphs, but still an optimistic bias is visible. It casts doubts on the much larger numbers of positives reported by *A*.

As a summary, we demonstrated firstly how previously used permutation methods *P *and *R *give overly optimistic results, and secondly, how also the more recent and more advanced autocorrelation method *A *has optimistic tendencies and better results can be obtained by the more conservative method *S*. The two multiple testing control methods had identical performance. Hence we can recommend the Benjamini-Hochberg method, as it is established and has a well documented theoretical basis, unlike the method used by Futschik and Herzel.

## Discussion

Microarray data is commonly noisy and can be plagued by effects not related to the biological question under study. It is therefore crucial to focus on null models of gene expression, *i.e*., what type of patterns we expect to see by chance. Typically most of the attention is given to careful formulation of the pattern we are looking for, such as formulation of a periodicity score. However, equal amount of attention should be given to formulation of the null model, *i.e*., defining what we expect to see when there are no patterns that we are interested in. Construction of traditional parametric statistical models for periodicity studies or any other complex and novel area of bioinformatics is very difficult. Randomization methods are an viable alternative for analytical significance testing.

However, simple randomization tests, as simple permutations applied to gene cycle data, do not produce meaningful information. Comparing any real data with noise will assess patterns in the data significant. So it is more important for a randomization test to retain the realistic general structure of the data than to remove all structure under study. Otherwise the result will be too optimistic.

Originally it was estimated that for 400 to 1000 genes the periodic signal is strong enough to be considered statistically significant [15]. In our analysis we found out that data contains evidence of periodicity only for a smaller number of genes, when using the same datasets as an evidence. This does not mean that there would be only a small number of cell cycle regulated genes, but that their robust identification requires more effort. A recent meta-analysis integrated large number of different datasets and reported more than 40 percent of genes in fission yeast to be periodically expressed [35]. That demonstrates how improving the data in quantity and quality allows to identify larger number of periodic genes. Besides increasing the number of timepoints, an obvious improvement in periodicity studies is to perform control experiments to get a good empirical null distribution and to use it in assessing the significance of results.

To stress the point, the seminal results of gene periodicity studies were reported in 1998, and now more than 10 years later studies are still made that change the interpretation of those results. Biosciences are a rapidly developing area of research with new data and instruments coming out at a fast pace, and therefore more attention should be placed on significance testing.

## Conclusions

When gene expression data is mined for complex patterns, little if no effort is made to test results for statistical significance. With randomization, significance testing can be extended from simple classical statistical tests to complex pattern mining. Existing methods for testing significance of periodic gene expression patterns were found too simplistic and optimistic. We introduced significance testing framework that accounts for multiple testing and allows simple yet more realistic null models to be crafted with randomization algorithms. As a result, a much smaller number of genes showed significant periodicity.

As DNA microarrays have now become mainstream and new high-throughput methods are rapidly being adopted, we argue that not only there will be need for data mining methods capable of coping with immense datasets, but there will also be need for a new approach to significance testing. We need methods that do not require demanding mathematical analytics and that can be easily modified and adopted to different situations. Randomization algorithms are intuitive and can be readily constructed by the growing numbers of computer literate bioresearchers. Future of biosciences seems to be characterized by large developments in data production capability and that development must be met with appropriate tools to guard against false interpretations.

## Authors' contributions

AK implemented the method and carried out experiments. NV carried out the mathematical analysis. AK and NV drafted the manuscript. The design and development of the methods was done by all authors. All authors have read and approved the final manuscript.

## Supplementary Material

Additional file 1 **Appendix: Periodicity score calculations**. Additional file 1 contains more detailed mathematical derivations of the results in section Analysis of periodicity score distributions. Click here for file
