# Supplementary material for: Randomization techniques for assessing the significance of gene periodicity results
Source: BMC Bioinformatics. 2011 Aug 9;12:330. doi: 10.1186/1471-2105-12-330 (PMC3199764; doi:10.1186/1471-2105-12-330)
Supplement: Additional file 1 — Appendix: Periodicity score calculations. Additional file 1 contains more detailed mathematical derivations of the results in section Analysis of periodicity score distributions. [file 1471-2105-12-330-S1.PDF]

Appendix to

Randomization techniques for assessing the significance of gene periodicity results

Aleksi Kallio, Niko Vuokko, Markus Ojala, Niina Haiminen and Heikki Mannila

In this Appendix we go through the calculations behind the results of periodicity score distribution analysis in more detail.

Let $A = (a_{gt})_{n\times m}$ be our data set and define function $c_k(x) = \sin\frac{2\pi kx}{m}$ that represents a perfect cyclical signal. The simple kth periodicity score for the gth row A_g of A is defined as

$$\mathcal{S}_k = \left(\frac{A_g \cdot c_k}{m}\right)^2 = \left(\frac{1}{m}\sum_{t=0}^{m-1} a_{gt}\sin\frac{2\pi kt}{m}\right)^2.$$

The following two standard equations will be useful going forward:

$$\sum_{t=0}^{m-1}\sin\frac{2\pi rt}{m}\sin\frac{2\pi st}{m} = \begin{cases} m/2 & \text{if } r=s \\ 0 & \text{otherwise}\end{cases}, \tag{1}$$

$$\sum_{t=0}^{m-1}\sin^4\frac{2\pi kt}{m} = \frac{3m}{8}. \tag{2}$$

Periodicity scores produced by P: Permuting all entries

Expected value of a periodicity score $\mathcal{S}_k$ in a fully randomized matrix is

$$\mathbb{E}[\mathcal{S}_k] = \frac{1}{n}\sum_{g=0}^{n-1}\frac{1}{(mn)!}\sum_{\sigma\in S_{mn}}\frac{1}{m!}\sum_{\tau\in S_m}\left(\frac{1}{m}\sum_{t=0}^{m-1} a_{\sigma(gm+t)}\sin\frac{2\pi k\tau(t)}{m}\right)^2,$$

where the mean has been taken over all rows, all matrix permutations and all signal permutations. The last operation over signal permutations is not necessary since it doesn't affect the result. It is done however, because it greatly simplifies the following manipulations.

By expanding the squared sum and moving the τ-sum inside we get

$$\mathbb{E}[\mathcal{S}_k] = \frac{1}{2nm^2}\sum_{g=0}^{n-1}\frac{1}{(mn)!}\sum_{\sigma\in S_{mn}}\sum_{t=0}^{m-1} a^2_{\sigma(gm+t)} = \frac{\mathbb{E}_{g,t}[a^2_{gt}]}{2m}.$$

Next we will calculate the variance of $\mathcal{S}_k$. We use the same procedure of exponent expansion and moving the τ-sum in:

$$\begin{aligned}\mathrm{Var}[\mathcal{S}_k] &= \frac{1}{n}\sum_{g=0}^{n-1}\frac{1}{(mn)!}\sum_{\sigma\in S_{mn}}\frac{1}{m!}\sum_{\tau\in S_m}\frac{1}{m^4}\left(\sum_{t=0}^{m-1} a_{\sigma(gm+t)}\sin\frac{2\pi k\tau(t)}{m}\right)^4 - \mathbb{E}[\mathcal{S}_k]^2 \\ &\overset{(1),(2)}{=} \frac{1}{nm^4}\sum_{g=0}^{n-1}\frac{1}{(mn)!}\sum_{\sigma\in S_{mn}}\left(\frac{-3}{4}\sum_{t=0}^{m-1} a^4_{\sigma(gm+t)} + 3\sum_{t,\ell} a^2_{\sigma(gm+t)}a^2_{\sigma(gm+\ell)}\right) - \mathbb{E}[\mathcal{S}_k]^2 \\ &= \frac{11}{4m^2}\mathbb{E}_{g,t}[a^2_{gt}]^2 - \frac{3}{4m^3}\mathbb{E}[a^4_{gt}].\end{aligned}$$

Periodicity scores produced by R: Permuting within rows

Expected value of a periodicity score $\mathcal{S}_k$ in a matrix randomized by rows is

$$\mathbb{E}[\mathcal{S}_k] = \frac{1}{n}\sum_{g=0}^{n-1}\frac{1}{m!}\sum_{\tau\in S_m}\left(\frac{1}{m}\sum_{t=0}^{m-1}a_{gt}\sin\frac{2\pi k\tau(t)}{m}\right)^2.$$

Doing the very same steps we easily end up again at the same result as with method P: $\mathbb{E}[\mathcal{S}_k] = \mathbb{E}_{g,t}[a_{gt}^2]/(2m)$. With this by-rows randomization method the variance however is different:

$$\begin{aligned}\operatorname{Var}[\mathcal{S}_k] &= \frac{1}{nm^4}\sum_{g=0}^{n-1}\frac{1}{m!}\sum_{\tau\in S_m}\left(\sum_{t=0}^{m-1}a_{gt}\sin\frac{2\pi k\tau(t)}{m}\right)^4 - \mathbb{E}[\mathcal{S}_k]^2\\ &\overset{(1),(2)}{=} \frac{1}{nm^4}\sum_{g=0}^{n-1}\left(\frac{-3}{4}\sum_{t=0}^{m-1}a_{gt}^4 + 3\sum_{t,\ell}a_{gt}^2a_{g\ell}^2\right) - \mathbb{E}[\mathcal{S}_k]^2\\ &= -\frac{3}{4m^3}\mathbb{E}_{g,t}[a_{gt}^4] + \frac{3}{m^2}\mathbb{E}_g[\mathbb{E}_t[a_{gt}^2]^2] - \frac{1}{4m^2}\mathbb{E}_{g,t}[a_{gt}^2]^2.\end{aligned}$$

Periodicity scores produced by A: Autocorrelation

In this randomization method the values of a data row $(X_t)_{1\times n}$ are generated as values of a martingale: $X_0 = Z_0, X_1 = \alpha X_0 + Z_1, X_2 = \alpha X_1 + Z_2, \ldots, X_{m-1} = \alpha X_{m-2} + Z_{m-1}$. We require $|\alpha| < 1$ and that the Z_t are i.i.d samples from some distribution $\mathcal{Z}$ with mean zero. Supposing that our data signal has the Fourier decomposition $\sum_k \alpha_k c_k$, we may calculate the signal's autocorrelation to equal

$$\alpha = \frac{\sum_k \alpha_k^2\cos\frac{2\pi k}{m}}{\sum_k \alpha_k^2}.$$

The expectation of cyclicity $\mathcal{S}_k = \left(\frac{X_i\cdot c_k}{m}\right)^2$ for the autocorrelation-based randomization can now be calculated by using the pairwise independence of Z_r at (a) and also some more complex trigonometric manipulations at (b):

$$\begin{aligned}\mathbb{E}[\mathcal{S}_k] &= \frac{1}{m^2}\mathbb{E}\left[\left(\sum_{j=0}^{n-1}\sin\frac{2\pi kj}{m}\sum_{r=0}^{j}\alpha^{j-r}Z_r\right)^2\right] \overset{(a)}{=} \frac{1}{m^2}\mathbb{E}\left[\sum_{r=0}^{m-1}\left(\frac{Z_r}{\alpha^r}\sum_{j=r}^{m-1}\alpha^j\sin\frac{2\pi kj}{m}\right)^2\right]\\ &= \frac{\operatorname{Var}(\mathcal{Z})}{m^2}\sum_{r=0}^{m-1}\left(\sum_{j=r}^{m-1}\alpha^{j-r}\sin\frac{2\pi kj}{m}\right)^2 \overset{(b)}{=} \frac{\operatorname{Var}(\mathcal{Z})}{2m\left(1-\alpha^2+2\alpha\cos\frac{2\pi k}{m}\right)} + \mathcal{O}\left(\frac{1}{m^2}\right),\end{aligned}$$

The variance can now be calculated based on the result for the mean:

$$\begin{aligned}\operatorname{Var}(\mathcal{S}_k) &= \frac{1}{m^4}\mathbb{E}\left[\left(\sum_{j=0}^{m-1}\sin\frac{2\pi kj}{m}\sum_{r=0}^{j}\alpha^{j-r}Z_r\right)^4\right] - \mathbb{E}[\mathcal{S}_k]^2\\ &= \left(\mathbb{E}[\mathcal{Z}^4] - 2\operatorname{Var}(\mathcal{Z})^2\right)\frac{1}{m^4}\sum_{r=0}^{m-1}\left(\sum_{j=r}^{m-1}\alpha^{j-r}\sin\frac{2\pi kj}{m}\right)^4 + \mathbb{E}[\mathcal{S}_k]^2\\ &= \mathbb{E}[\mathcal{S}_k]^2 + \mathcal{O}\left(\frac{1}{m^3}\right).\end{aligned}$$

Periodicity scores produced by S: Splitting rows into parts

We simplify the analysis with an assumption that the cyclicity of the genes stays similar within the samples. This is acceptable since both methods A and S make this assumption implicitly in their model for the data. We denote the kth Fourier coefficient of the gth gene with α_{gk}, *i.e.*, the gth gene has Fourier decomposition $e_{gt} = \sum_k \alpha_{gk} c_k(t)$. We can now easily calculate the expectation of cyclicity for any randomized row of the data:

$$\mathbb{E}[\mathcal{S}_k] = \frac{1}{n^2} \sum_{g=0}^{n-1} \sum_{h=0}^{n-1} \left(\frac{\alpha_{gk}}{4} + \frac{\alpha_{hk}}{4} \right)^2 = \frac{\mathbb{E}_g[\alpha_{gk}^2] + \mathbb{E}_{g,h}[\alpha_{gk}\alpha_{hk}]}{8}.$$

Also the variance can be straightforwardly calculated:

$$\mathrm{Var}[\mathcal{S}_k] = \frac{1}{n^2} \sum_{g=0}^{n-1} \sum_{h=0}^{n-1} \left(\frac{\alpha_{gk}}{4} + \frac{\alpha_{hk}}{4} \right)^4 = \frac{\mathbb{E}_g[\alpha_{gk}^4] + 3\mathbb{E}_{g,h}[\alpha_{gk}^2\alpha_{hk}^2] + 4\mathbb{E}_{g,h}[\alpha_{gk}^3\alpha_{hk}]}{128}.$$
